# Concurrence of Juvenile Idiopathic Arthritis and Multiple Sclerosis

**DOI:** 10.1155/2011/162857

**Published:** 2012-01-04

**Authors:** Ben Abdelghani Kaouther, Souabni Leila, Belhadj Salwa, Zakraoui Leith

**Affiliations:** Rheumatology Department, Mongi Slim University Hospital and University of Tunis El Manar, 2034 La Marsa, Tunisia

## Abstract

We report a 21-year-old female patient known to have Juvenile idiopathic arthritis (JIA) who later developed multiple sclerosis (MS). The disease was documented on the brain and cerebral magnetic resonance imaging (MRI) and the visual evoked potential. Our case emphasizes the need to evaluate the symptoms and brain MRI carefully. The concurrence of MS and JIA is uncommon. The possible relationship between the 2 diseases was discussed.

## 1. Introduction

Juvenile idiopathic arthritis (JIA) is a systemic pathology of connective tissue characterized by a chronic inflammatory process with an autoimmune background, whereas multiple sclerosis (MS) is a demyelination disease with an important role of immune disorders in its pathogenesis. The concurrence of these two diseases is not common. 

## 2. Case Report

**Figure 1:**
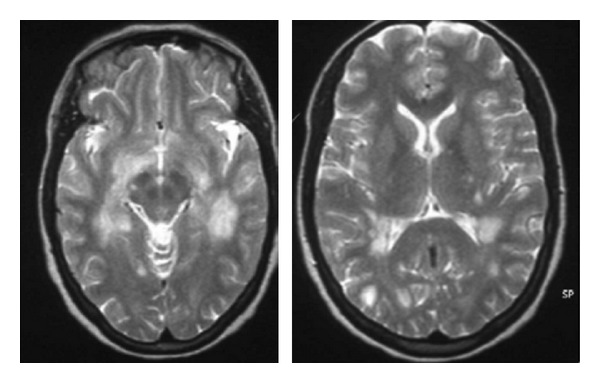
Brain MRI revealed hyperintense lesions of white matter on axial T2-weighted images.

We described a 21-year-old woman with a five-year history of polyarticular seropositive and erosive JIA, treated with leflunomide 20 mg daily. She presented with problems in balance and disequilibrium sensation. She did not have a history of vaccination or viral infection. Decreases in sensitivity to light touch were noted in the right trigeminal area. A diplopia on lateral gaze and a Romberg sign was noted. Magnetic resonance imaging of the brain and spinal cord showed multiple areas of increased signal intensity involoving the deep white matter (Figure 1). Spinal fluid evaluation showed selective increase in immunoglobulin G. Bacterial and viral studies were negative. Visual evoked potential showed retrobulbar optic neuritis. The presumptive diagnosis was multiple sclerosis. The patient was started on intravenous methylprednisolone. She improved significantly with partial resolution of her disequilibrium.

## 3. Discussion

The association of MS and JIA is not frequently described. Only one case had been already reported [1]. Some observations of association with adult chronic arthritis have been described. Toussirot et al. [2] have reported 14 cases of association of rheumatoid arthritis (RA) and MS. In another study including 898 Chinese patients with MS and 4490 randomly matched controls, patients with MS were more likely to have RA [3]. This association is certainly not hazardous. The MS is a T-cell-mediated autoimmune disease similar to JIA with genetic and environmental factors playing a role in their pathogenesis. Certain viruses like Epstein Barr virus have been incriminated as a possible etiologic factor in both diseases. Another relevant finding is the citrullination phenomenon, which is already involved in RA has been also involved in MS [4]. Indeed, extensive deimination of brain proteins is observed in active lesions of MS. This deimination is catalyzed by the peptidylarginine deiminases (PADs) and some alleles of its gene confer susceptibility to RA [5]. The relationship between JIA and MS is strengthened by reports of demyelinating events occurring in the disease course of patients receiving tumor necrosis factor TNF-*α* antagonists [6]. Robinson had hypothesized that neurological events could be caused by latent neurologic disease unmasked by anti-TNF blockers or by preexisting neurologic disease [7]. However, in a recent study, data suggested reduced comorbidity between MS and RA. This novel finding undermines the assertion that cases of MS among patients treated with TNF inhibitors are simply coincidental. Since the study period predated availability of TNF inhibitors, the results were not confounded by this parameter, providing a unique opportunity to characterize baseline rates of coexistence in a population of RA and MS patients naive to this type of biologic therapy [8].

## 4. Conclusion

Our case emphasizes the need to evaluate the neurologic symptoms carefully when using TNF-*α* inhibitors in JIA.

##  Conflict of Interests 

The authors declare that there is no conflict of interests.
